# Supplementary material for: Whole-brain dynamical modelling for classification of Parkinson’s disease
Source: Brain Commun. 2022 Dec 15;5(1):fcac331. doi: 10.1093/braincomms/fcac331 (PMC9798283; doi:10.1093/braincomms/fcac331)
Supplement: fcac331_Supplementary_Data [file fcac331_supplementary_data.pdf]

Supplementary material

Simulated BOLD signals

The neurovascular coupling describes that the changes of the induced signals $s(t)$ driven by the EPSP input link to the changes in the cerebral blood flows (CBF) $f(t)$ as the blood inflow

$$\dot{s}(t) = -\kappa s(t) - \gamma(f(t) - 1) + \frac{y_{EPSP}(t)}{A}, \tag{1}$$

$$\dot{f}(t) = s(t). \tag{2}$$

Equations 1 and 2 govern the dynamics of the induced signal and CBF, respectively. Parameters κ and γ are the rate constants that regulate ultra-slow endogenous fluctuations at around 0.09 Hz.[1] The normalized neural response, i.e., $y_{EPSP}(t)$ divided by the amplitude A of the parameter in the electrical model, drives the induced slow fluctuation. Consequently, CBF signals simultaneously influence the changes of the cerebral blood volume (CBV) $v(t)$ and deoxyhemoglobin content (DOH) $q(t)$ as described by the following equations:

$$\dot{v}(t) = \frac{1}{t_{MTT}}[f(t) - f_{out}(v,t)], \tag{3}$$

$$\dot{q}(t) = \frac{1}{t_{MTT}}\left[f(t)\frac{E(f)}{E_0} - f_{out}(v,t)\frac{q(t)}{v(t)}\right]. \tag{4}$$

The mean transit time t_{MTT} scales both differential equations for passing a bolus of the blood through the vein. To estimate CBV changes, Equation 3 models a difference between the blood inflow $f(t)$ and the blood outflow $f_{out}(v,t)$. Subsequently, we can calculate the changes of DOH using the dynamics of CBF and CBV by regarding oxygen extraction fraction $E(f)$ in Equation 4. Parameter E_0 is the net oxygen extraction fraction at rest,

$$f_{out}(v) = v^{1/\alpha}, \tag{5}$$

$$E(f) = 1 - (1 - E_0)^{1/f}. \tag{6}$$

Equation 5 provides the relationship between CBF and CBV, where Grubb *et al.*[2] empirically found α is 0.38. Equation 6 is a non-linear function of CBF, and describes an effect of CBF on the oxygen extraction fraction, see the reference[1] for details. Using CBV and DOH, we can calculate simulated BOLD signals y_{BOLD}:

$$y_{BOLD} = V_0\left[k_1(1-q) + k_2\left(1 - \frac{q}{v}\right) + k_3(1-v)\right], \tag{7}$$

where V_0 is the resting blood volume fraction, and parameters k_1, k_2, and k_3 depend on the magnetic field strength as follows:

$$k_1 = 4.3 \cdot \vartheta_0 \cdot E_0 \cdot TE, \qquad k_2 = \varepsilon \cdot r_0 \cdot E_0 \cdot TE, \qquad k_3 = 1 - \varepsilon. \tag{8}$$

Parameters ϑ_0, TE, ε, and r_0 are the frequency offset for 3 T scanner, the echo time, the ratio of intra/extra-vascular signal, and the sensitivity of changes in intra-vascular signal relaxation rate with changes in oxygen saturation, respectively.[1] The parameter values of the BW model for BOLD signals are given in Table 2.

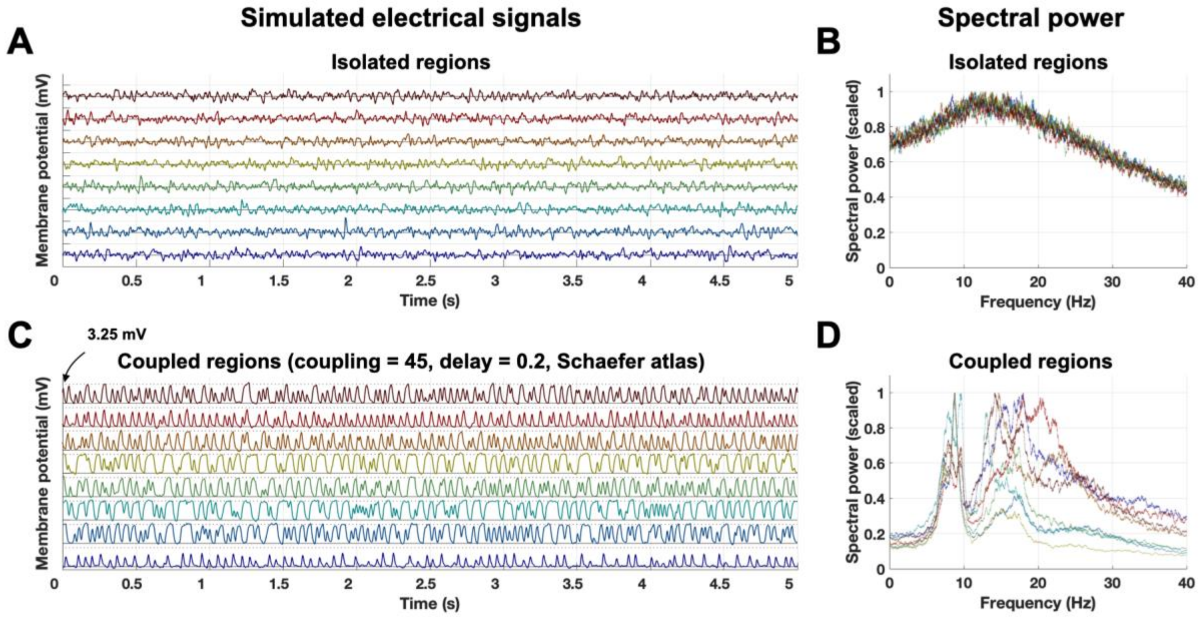

Supplementary Figure 1. Examples of **(A, C)** time courses of the simulated excitatory post-synaptic potentials (EPSP) and **(B, D)** their spectral power distributions of a few brain regions for **(A, B)** isolated (global coupling = 0) and **(C, D)** coupled cases for the Schaefer atlas. In the latter case, global coupling = 45 and global delay = 0.2 are the optimal model parameters of the neuroimaging model fitting. The peaks of the maximal spectral power for the isolated regions in (B) are around 13 Hz. The dotted horizontal lines in (C) indicate the maximum EPSP (3.25 mV), which is the specified value as the maximal EPSP kernel in Table 2.

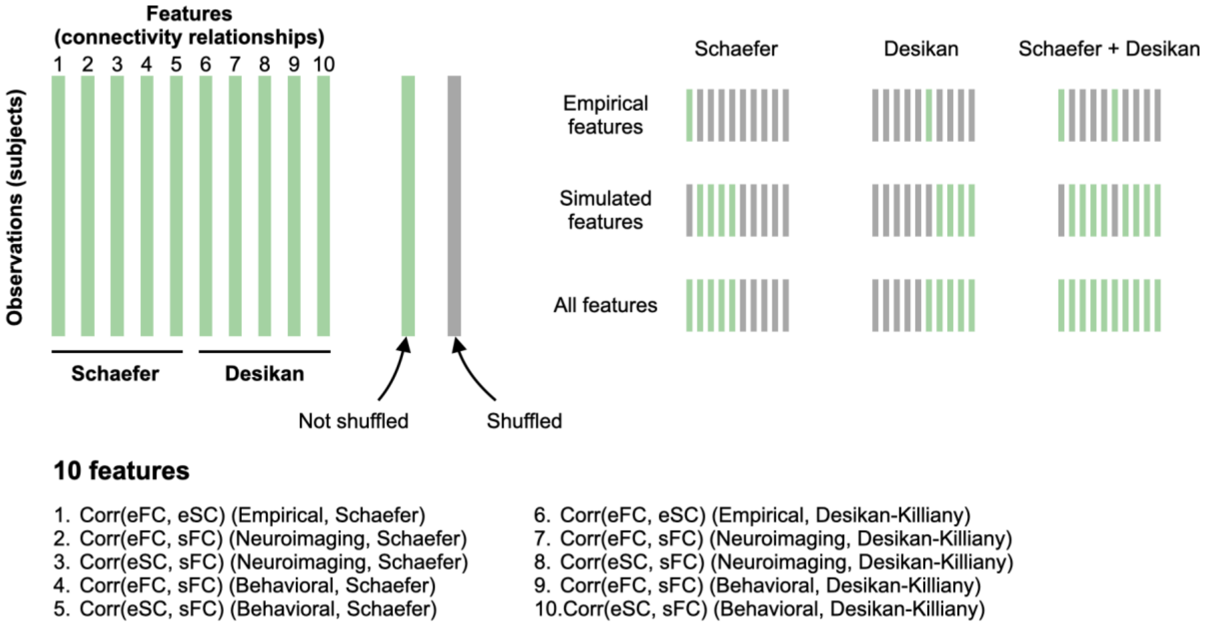

Supplementary Figure 2. Features and feature conditions used for PD classification. Ten connectivity relationships listed in the plot were used during machine-learning training and testing for PD classification as features. To investigate the impact of simulated results on the prediction performance, we considered 9 feature conditions as illustrated in the right part of the plot. Here, a few features of interest were selected (green bars), while the other features were randomly shuffled across subjects (gray bars). Shuffling is done for each feature separately, i.e., shuffling within a feature gives the same distribution of the values but randomized feature values across subjects, which destroys the correspondence between the (brain) feature and behavioral labels (PD or HC). The shuffled feature is supposed to not contribute to classification performance, but we always keep the same number (ten) of features in all feature conditions of the machine-learning experiments. Abbreviations: FC = functional connectivity; PD = Parkinson’s disease; SC = streamline count.

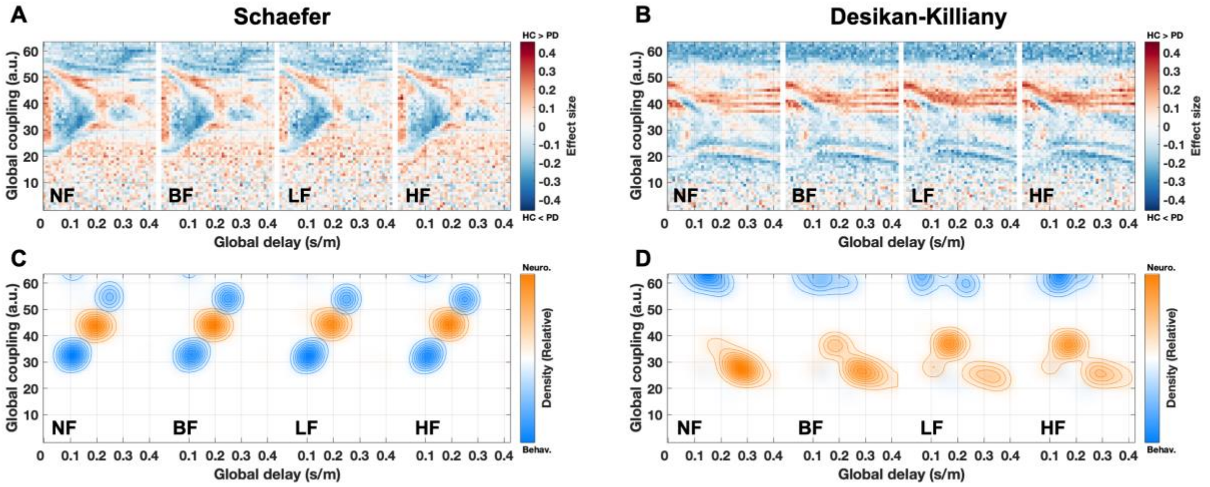

Supplementary Figure 3. Parameter maps of the effect size of the difference of eSC-sFC correlation values between PD and HC subject groups used for the behavioral model fitting. The filtering conditions are indicated in the plots for **(A)** the Schaefer atlas and **(B)** the Desikan-Killiany atlas. Effect sizes in the (τ_{global}, C)-parameter plane were calculated by a non-parametric Wilcoxon rank-sum two-tailed test between HC and PD subject groups in the eSC-sFC correlation values for each parameter point. **(C, D)** Distributions of optimal parameters derived from the neuroimaging model fitting (orange, all subjects, n=116) and the behavioral model fitting (blue, repeated sub-sampling, n=1000) for **(C)** the Schaefer atlas and **(D)** the Desikan-Killiany atlas. Abbreviations: PD = Parkinson's disease; HC = healthy controls; NF = no filtering; BF = broad band ([0.01,0.1] Hz); LF = low-frequency band ([0.01,0.05] Hz); HF = high-frequency band ([0.05,0.1] Hz).

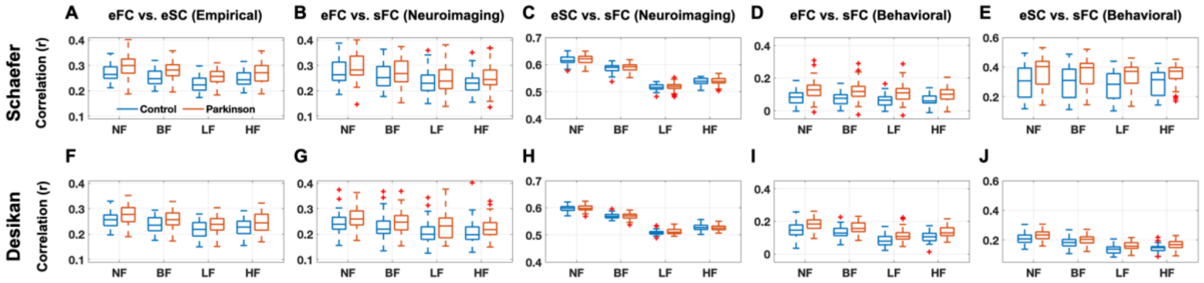

Supplementary Figure 4. Comparison of connectivity correspondences between HC and PD subject groups as reflected by the connectivity relationships of empirical and simulated results for **(A-E)** the Schaefer atlas and **(F-J)** the Desikan-Killiany atlas for **(A, F)** the empirical structure-function relationship (eFC vs. eSC), **(B, C, G, H)** functional (eFC vs. sFC) and structure-function (eSC vs. sFC) relationships for the neuroimaging model fitting, and **(D, E, I, J)** connectome relationships (eFC vs. sFC and eSC vs. sFC) for the behavioral model fitting. Abbreviations: NF = no filtering; BF = broad band ([0.01,0.1] Hz); LF = low-frequency band ([0.01,0.05] Hz); HF = high-frequency band ([0.05,0.1] Hz); FC = functional connectivity; SC = streamline count.

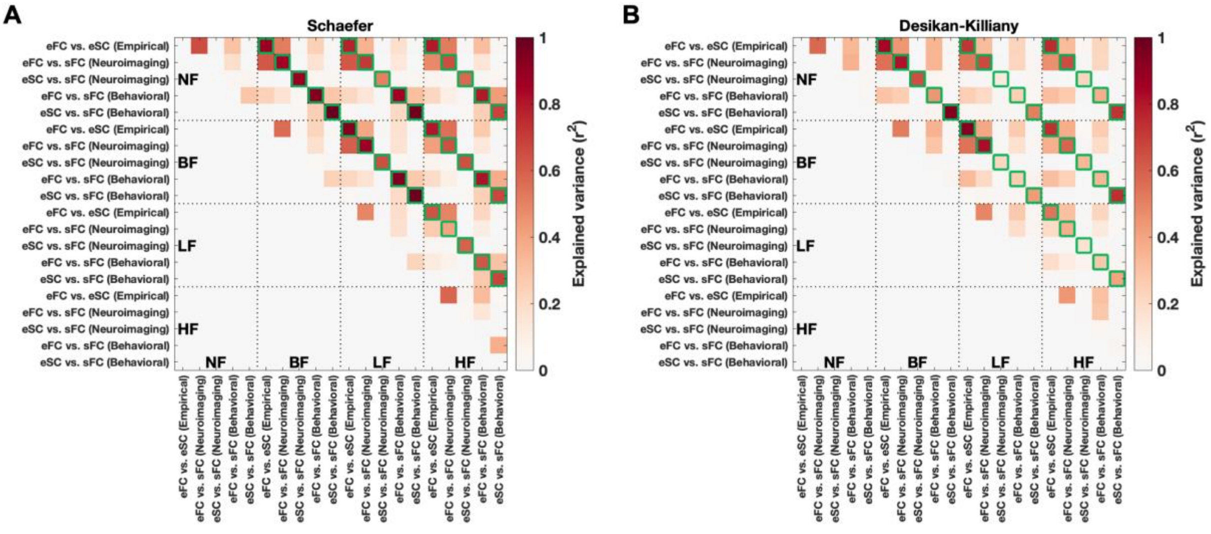

Supplementary Figure 5. Explained variances (EV, squared correlation r^2) between five connectivity relationships for **(A)** the Schaefer atlas and **(B)** the Desikan-Killiany atlas. The five connectivity relationships are corr(eSC, eFC) (empirical), corr(eFC, sFC) (neuroimaging), corr(eSC, sFC) (neuroimaging), corr(eFC, sFC) (behavioral), and corr(eSC, sFC) (behavioral). Due to the four considered temporal filtering conditions of NF, BF, LF, and HF indicated in the plots, the intra-/inter-condition EVs were obtained using 20 connectivity relationships (see the axes). The green boxes are for the same types of connectivity relationships under different filtering conditions. Abbreviations: NF = no filtering; BF = broad band ([0.01,0.1] Hz); LF = low-frequency band ([0.01,0.05] Hz); HF = high-frequency band ([0.05,0.1] Hz).

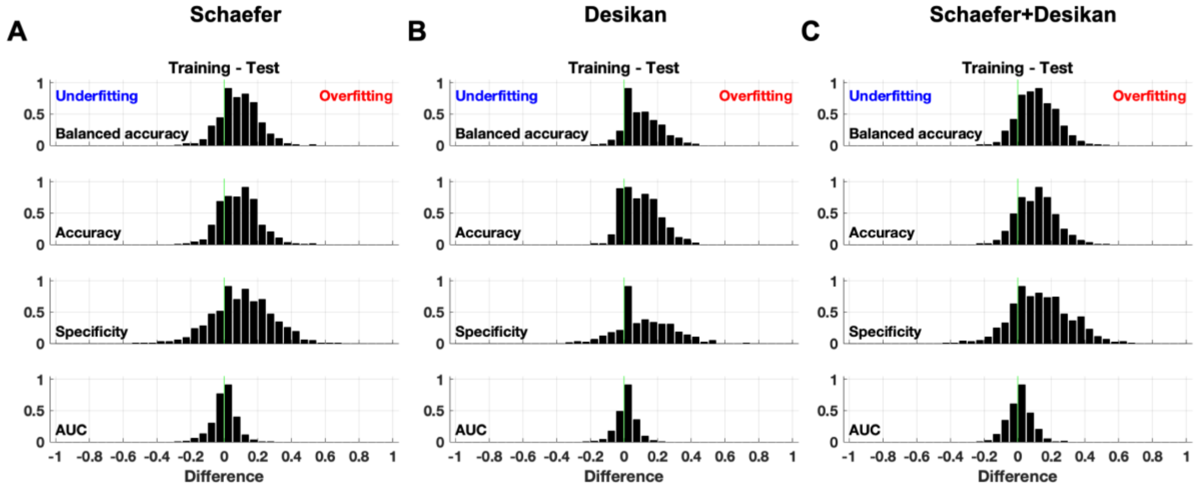

Supplementary Figure 6. Differences of model performance between training and test sets (Training – Test) for PD prediction including all filtering conditions and all features for the **(A)** Schaefer atlas, **(B)** Desikan-Killiany atlas, and **(C)** multiple atlases, i.e., the Schaefer and Desikan-Killiany atlases. The considered performance measures are indicated in the plots. The green vertical lines indicate zero differences. The positive differences are overfitting cases, and the negative ones are underfitting. Abbreviation: AUC = area-under-curve.

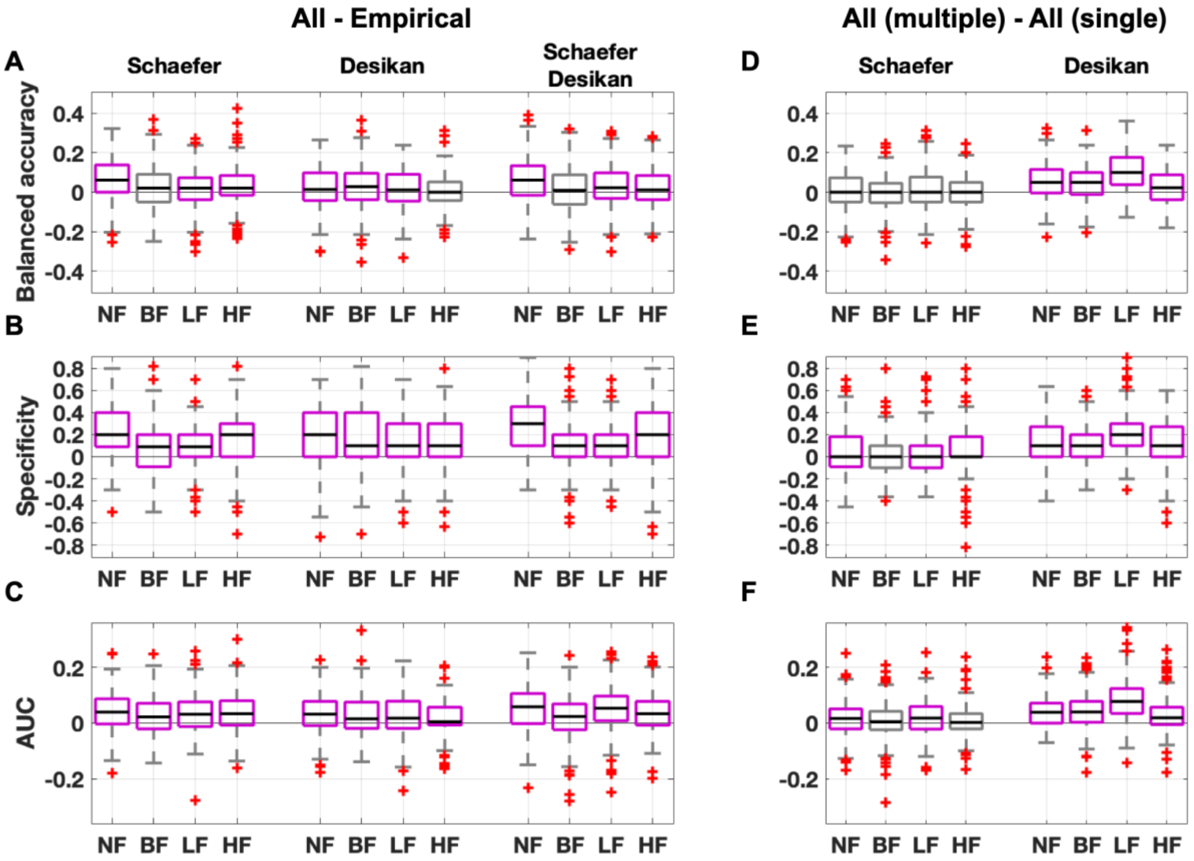

Supplementary Figure 7. Comparisons of the prediction performance between the feature conditions used for the subject classification, see Supplementary Figure 2. The differences in the performance measures are illustrated as box plots for (A-C) "All features" versus "Empirical features" (All - Empirical) for the Schaefer, Desikan-Killiany, and multiple (Schaefer and Desikan-Killiany) atlases as indicated on the top of the plot (A) and for **(D-F)** "All features" for the multiple atlases versus "All features" for single atlases (All(multiple) – All (single)) as indicated on top of plot (D). The performance measures are **(A, D)** balanced accuracy, **(B, E)** specificity, and **(C, F)** AUC of ROC curves. The filtering conditions are given on the horizontal axes. The purple boxes depict significantly different performance (Wilcoxon signed-rank two-tail test and Bonferroni corrected $p < .05$). Abbreviations: NF = no filtering; BF = broad band ([0.01,0.1] Hz); LF = low-frequency band ([0.01,0.05] Hz); HF = high-frequency band ([0.05,0.1] Hz).

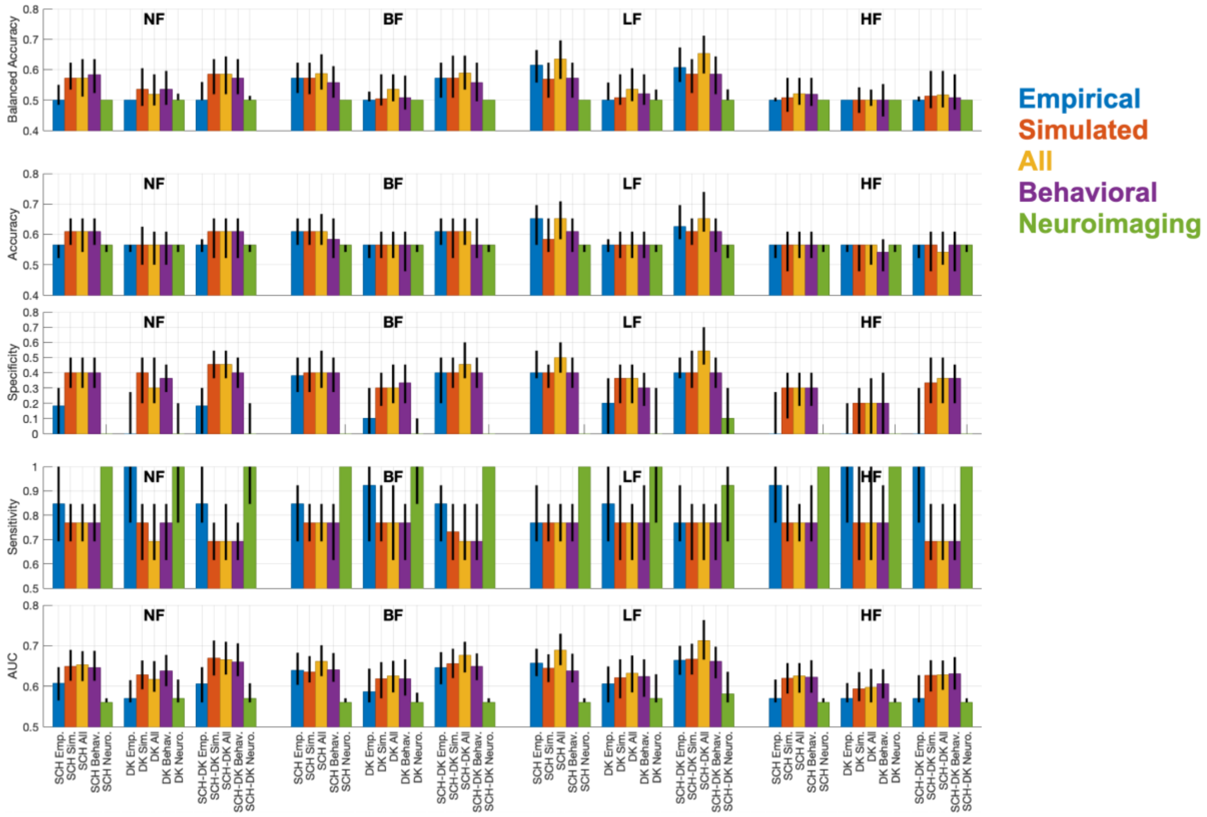

Supplementary Figure 8. Prediction performance as given by the balanced accuracy, accuracy, specificity, sensitivity, and AUC of ROC curves using optimal simulated connectomes (corresponding to the optimal model parameters) from the behavioral fitting only (purple) and from the neuroimaging fitting only (green) as additional feature conditions to those presented in Supplementary Figure 2 (also depicted here for comparison, see the legend). The error bars indicate interquartile ranges, and the heights of bars are the medians. The filtering conditions are indicated in the plots. Abbreviations: NF = no filtering; BF = broad band ([0.01,0.1] Hz); LF = low-frequency band ([0.01,0.05] Hz); HF = high-frequency band ([0.05,0.1] Hz); SCH = Schaefer; DK = Desikan-Killiany, Emp. = Empirical features, Sim. = Simulated features, All = All features, Neuro. = Simulated features from the neuroimaging model fitting only, and Behav. = Simulated features from the behavioral model fitting only.

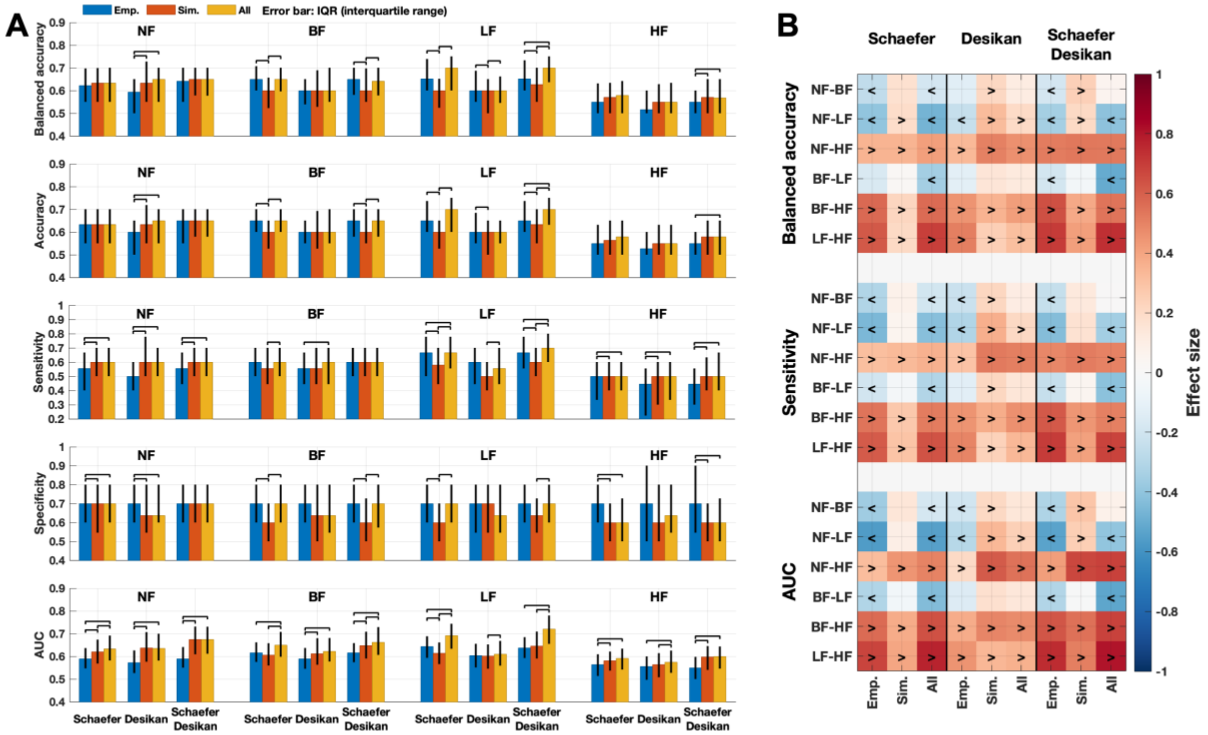

Supplementary Figure 9. Summary of the performance of PD classification using the three different feature conditions: empirical features (blue bars), simulated features (red bars), and all features (yellow bars) for the balanced subject configuration (n=99, Supplementary Table 1) controlled for the balanced age and sex and size of subject groups (HC versus PD). **(A)** Median values of the balanced accuracy, accuracy, sensitivity, specificity and area-under-curve (AUC) of the receiver operating characteristics (ROC) curves for all considered parcellations and filtering conditions are shown in each panel. The error bars indicate the interquartile range across iterations of the outer loop of the nested cross-validation procedure (see Fig. 2 in the main text). The black lines connecting two conditions indicate significantly different performance between feature conditions. **(B)** Effect sizes between filtering conditions for each feature condition. The signs '<' and '>' indicate which condition is significantly larger than the other. For example, '<' sign for 'NF-LF' indicated on the vertical axes means NF < LF for a given performance indicated on the horizontal axes. The Wilcoxon signed-rank two-tail test was used for comparisons across feature and filtering conditions (Bonferroni corrected statistics). Abbreviations: PD = Parkinson's disease; NF = no filtering; BF = broad band ([0.01,0.1] Hz); LF = low-frequency band ([0.01,0.05] Hz; HF = high-frequency band ([0.05,0.1] Hz).

Supplementary Table 1 Demography of a balanced subject configuration (excluding 17 oldest patients from 116 subjects).

Groups	Mean (standard deviation) years		Statistical tests	*p*-values
	**All subjects**		**Chi-square goodness-of-fit test**	
All	56.62 (9.24)		99 subjects	0.235
	**Healthy controls**	**Patients**	**Wilcoxon rank-sum two-tail test**	
All	55.02 (9.69)	58.31 (8.42)	51 healthy controls versus 48 patients	0.062
Female	56.52 (9.40)	60.80 (8.96)	21 healthy controls versus 20 patients	0.201
Male	53.97 (9.74)	56.54 (7.53)	30 healthy controls versus 28 patients	0.156
	**Females**	**Males**	**Wilcoxon rank-sum two-tail test**	
All	58.61 (9.43)	55.21 (8.84)	41 females versus 58 males	0.095

References

1. Havlicek M, Roebroeck A, Friston K, Gardumi A, Ivanov D, Uludag K. Physiologically informed dynamic causal modeling of fMRI data. *NeuroImage.* 2015;122:355-372.

2. Grubb RL, Jr., Raichle ME, Eichling JO, Ter-Pogossian MM. The effects of changes in PaCO2 on cerebral blood volume, blood flow, and vascular mean transit time. *Stroke.* 1974;5(5):630-639.
